# Pretransplant Locoregional Therapy for Hepatocellular Carcinoma: Evaluation of Explant Pathology and Overall Survival

**DOI:** 10.3389/fonc.2016.00143

**Published:** 2016-06-13

**Authors:** Eliza W. Beal, Kristin M. Dittmar, A. James Hanje, Anthony J. Michaels, Lanla Conteh, Gail Davidson, Sylvester M. Black, P. Mark Bloomston, Mary E. Dillhoff, Carl R. Schmidt

**Affiliations:** ^1^Department of Surgery, The Ohio State University Wexner Medical Center, James Cancer Hospital, Columbus, OH, USA; ^2^Department of Radiology, The Ohio State University Wexner Medical Center, James Cancer Hospital, Columbus, OH, USA; ^3^Division of Gastroenterology, Hepatology and Nutrition, Department of Internal Medicine, The Ohio State University Wexner Medical Center, Columbus, OH, USA

**Keywords:** liver transplantation, carcinoma, hepatocellular, chemoembolization, therapeutic, radiofrequency ablation, microwave ablation, locoregional bridging therapy

## Abstract

**Background and objectives:**

Liver transplant is an important treatment option for patients with hepatocellular carcinoma (HCC) within Milan criteria. We sought to determine the rate of complete tumor necrosis after bridging therapy.

**Methods:**

The medical records of all 178 patients undergoing liver transplantation between January 1, 2008 and July 31, 2015 were reviewed. Response to therapy by imaging was based on mRECIST criteria (1).

**Results:**

Sixty-three (35%) patients had HCC. Forty-three (68%) were treated with at least one bridging therapy and 14 (22%) were diagnosed incidentally. Eighteen (42%) underwent TACE and 25 (58%) underwent ablation. Twenty (80%) patients who underwent ablation and nine (60%) who underwent TACE had complete response based on imaging. Viable tumor was identified in explant pathology in 32 patients (74%). The presence or absence of viable tumor was not associated with overall survival.

**Conclusion:**

Rates of viable tumor based on pathologic analysis in the hepatic explant were high after bridging therapy, but not associated with worse outcome. We conclude that serial bridging to achieve complete pathologic tumor response is not needed prior to transplant for HCC, and presence of complete response by imaging is adequate. Further studies are needed to determine if cancer cells that appear viable are alive.

## Introduction

Hepatocellular carcinoma (HCC) is one of the few cancers with increasing incidence in the United States over the past two decades (2–4). Orthotopic liver transplant is widely accepted as a potentially curative treatment option for patients with early stage cancer defined by the Milan criteria (5–8). One limitation of this therapy is the growing incidence of waitlist dropouts due to increasing time on the list related to limited organ availability (9). In a recent consensus statement published in the *Journal of the National Comprehensive Cancer Network* (JNCCN), it is recommended that patients with HCC be considered for bridging therapy (10) if they are expected to wait more than 6 months for transplantation in order to reduce dropouts from the waitlist secondary to tumor progression (11).

The most common bridging therapy options we use are transarterial chemoembolization (TACE) and percutaneous or laparoscopic ablation (11). Reported rates of complete tumor necrosis, or complete pathologic response, vary based on treatment modality with response rates between 0 (12) and 57% (13) reported for TACE and 8.3 (12) and 74% (14) reported for radiofrequency ablation. The primary objective of this study was to determine the rate of complete tumor necrosis after locoregional bridging therapy for HCC prior to liver transplantation, as determined by explant histopathology.

## Materials and Methods

After Institutional Review Board approval, the records of all adult patients who underwent liver transplantation between January 1, 2008 and July 31, 2015 were reviewed using the electronic medical record and a prospectively maintained transplantation database. Demographic variables were collected, including type of transplant, age, gender, calculated Model for End-stage Liver Disease (MELD) score, body mass index (BMI), etiology of disease, and medical comorbidities. MELD score was calculated using the following formula: 10 × {[0.957 × ln(creatinine)] + [0.378 × ln(bilirubin)] + [1.12 × ln(INR)]} + 6.43, and exception points were not added. For any value <1.0, the value was set to 1.0 to avoid negative results. Any patient who had received dialysis twice in the previous 7 days had creatinine set to 4.0.

All patients in this cohort had a cancer therapy plan discussed at our center’s multidisciplinary liver tumor board. In general, ablation is considered for patients with three or less tumors with size <4 cm for any single tumor. Patients treated with at least one TACE prior to transplant were given either a triple-drug cocktail (mitomycin C, cisplatin, and doxorubicin) with Lipiodol or DC beads^®^. TACE procedures were performed by our interventional radiology team using percutaneous femoral access and fluoroscopic guidance. Ablations were either performed laparoscopically or percutaneously using CT-guidance. Laparoscopic ablations were performed by either of two surgical oncologists using a microwave source. CT-guided ablations were performed using radiofrequency ablation or microwave ablation and were performed by an interventional radiologist. The majority of patients were treated with appropriate antiviral therapy until the time of transplant.

All liver explants were examined by a pathologist specializing in hepatobiliary disease. Tumor viability was determined using standard hematoxylin and eosin staining. The presence and extent of tumor necrosis was characterized based on an estimated percent of necrosis. Cases where there was doubt about complete response were placed in the viable tumor category for the purpose of analysis. Statistical analysis was performed using Statistical Package for the Social Sciences version 22 (IBM, Armonk, NY, USA). Categorical variables were compared using Fisher’s exact test, and continuous variables were compared using the independent sample *T*-test. Survival curves were computed with the Kaplan–Meier method and differences in outcome determined by log-rank test.

## Results

There were 178-liver transplants completed during the study period, including 9-combined liver–kidney transplants (Table 1). The most common etiologies leading to transplantation were cirrhosis related to hepatitis C virus (HCV) or hepatitis B virus (HBV) infection, alcohol abuse, and non-alcoholic steatohepatitis. Sixty-three (35%) patients had HCC. Fourteen (22%) of these were diagnosed incidentally on explant pathology. Patients transplanted with HCC were older, with lower natural MELD and higher proportion of HCV and HBV infections, and more often had a history of prior abdominal surgery. Forty-three (68%) of the 63 patients with HCC were treated with at least one bridging therapy prior to transplantation, and all patients were within Milan criteria (and none were downstaged). Eighteen (42%) of these patients underwent TACE initially and 25 (58%) underwent ablation. Eleven patients underwent a second bridging procedure – 10 TACE and 1 laparoscopic microwave ablation.

**Table 1:** **Descriptive characteristics of entire cohort, HCC group, and non-HCC group**.

	Entire cohort (*n* = 178)	HCC (*n* = 63)	Non-HCC (*n* = 115)		HCC, bridged (*n* = 43)	HCC, non-bridged (*n* = 20)		TACE (*n* = 18)	Ablation (*n* = 25)	
Liver transplant only	169 (94.9%)	59 (93.7)	110 (95.7%)	*p* = 0.560	42 (97.7%)	17 (85.0%)	*p* = 0.055	18 (100.0%)	24 (96.0%)	*p* = 0.541
Gender male	123 (69.1%)	43 (68.1%)	80 (69.6%)	*p* = 0.856	31 (72.1%)	12 (60.0%)	*p* = 0.337	13 (72.2%)	18 (72.0%)	*p* = 0.888
Age at transplant (mean)	53.14	56.33	51.39	*p* = 0.001	56.42	56.15	*p* = 0.903	57.8	55.44	*p* = 0.466
Age at transplant (median)	55	57	53	*p* = 0.002	57	56	*p* = 0.830	56.5	58	*p* = 0.458
Calculated MELD at transplant (mean)	21.87	16.73	24.69	*p* = 0.000	13.83	22.98	*p* = 0.000	13.2	14.72	*p* = 0.820
Pre-transplant AFP	20.09	40.84	7.55	*p* = 0.020	55.59	8.24	*p* = 0.225	39.99	67.29	*p* = 0.607
BMI (mean)	29.8	30	29.75	*p* = 0.795	29.8	30.44	*p* = 0.677	29.6	29.93	*p* = 0.864
Etiology of cirrhosis										
Hepatitis C virus	73 (41%)	42 (66.7%)	31 (27%)	*p* = 0.000	28 (65.1%)	14 (70.0%)	*p* = 0.702	10 (55.6%)	18 (72.0%)	*p* = 0.001
Hepatitis B virus	7 (3.9%)	6 (9.5%)	1 (0.9%)	*p* = 0.005	5 (11.6%)	1 (5.0%)	*p* = 0.404	2 (11.1%)	3 (12.0%)	*p* = 0.012
Alcoholic	38 (21.3%)	11 (17.5%)	27 (23.5%)	*p* = 0.349	6 (14.0%)	5 (25.0%)	*p* = 0.282	3 (16.7%)	3 (12.0%)	*p* = 0.371
Non-alcoholic steatohepatitis	24 (13.5%)	7 (11.1%)	17 (14.8%)	*p* = 0.493	4 (9.3%)	3 (15.0%)	*p* = 0.503	3 (16.7%)	1 (4.0%)	*p* = 0.318
Alpha-1 antitrypsin deficiency	5 (2.8%)	2 (3.2%)	3 (2.6%)	*p* = 0.827	1 (2.3%)	1 (5.0%)	*p* = 0.573	0 (0.0%)	1 (4.0%)	*p* = 0.718
Cryptogenic	13 (7.3%)	1 (1.6%)	12 (10.4%)	*p* = 0.030	0 (0.0%)	1 (5.0%)	*p* = 0.139	0 (0.0%)	0 (0.0%)	N/A
Acute liver failure	3 (1.7%)	0 (0%)	3 (2.6%)	*p* = 0. 196	0 (0.0%)	0 (0.0%)	N/A	0 (0.0%)	0 (0.0%)	N/A
Primary sclerosing cholangitis or primary biliary cirrhosis	15 (8.4%)	0 (0%)	15 (13%)	*p* = 0.003	0 (0.0%)	0 (0.0%)	N/A	0 (0.0%)	0 (0.0%)	N/A
Others (*n*, %)	20 (11.2%)	2 (3.2%)	18 (15.7%)	*p* = 0.012	0 (0.0%)	2 (10.0%)	*p* = 0.035	0 (0.0%)	0 (0.0%)	N/A
Medical comorbidities										
Diabetes mellitus	45 (25.3%)	15 (23.8%)	30 (26.1%)	*p* = 0.738	11 (25.6)	4 (20.0%)	*p* = 0.628	4 (22.2%)	7 (28.0%)	*p* = 0.910
Prior abdominal surgery	88 (49.4%)	39 (61.9%)	49 (42.6%)	*p* = 0.041	26 (60.5%)	13 (65.0%)	N/A	9 (50.0%)	17 (68.0%)	*p* = 0.365
Hypertension	64 (36%)	22 (34.9%)	42 (36.%)	*p* = 0.831	13 (30.2%)	9 (45%)	*p* = 0.252	5 (27.8%)	8 (32.0%)	*p* = 0.642
Coronary artery disease	12 (6.7%)	5 (7.9%)	7 (6.1%)	*P* = 0.638	4 (9.3%)	1 (5.0%)	*P* = 0.556	3 (16.7%)	3 (12.0%)	*p* = 0.686
End-stage renal disease	8 (4.5%)	2 (3.2%)	6 (5.2%)	*p* = 0.529	0 (0.0%)	2 (10.0%)	*p* = 0.035	0 (0.0%)	0 (0.0%)	*p* = 0.263
Atrial fibrillation	4 (2.2%)	0 (0.0%)	4 (3.5%)	*p* = 0.134	0 (0.0%)	0 (0.0%)	N/A	0 (0.0%)	0 (0.0%)	N/A
COPD	8 (4.5%)	4 (6.3%)	4 (3.5%)	*p* = 0.377	2 (4.7%)	2 (10.0%)	*p* = 0.418	1 (5.6%)	1 (4.0%)	*p* = 0.969
History of arrhythmia (not A. Fib.)	2 (1.1%)	1 (1.6%)	1 (0.9%)	*p* = 0.664	1 (2.3%)	0 (0.0%)	*p* = 0.492	0 (0.0%)	1 (4.0%)	*p* = 0.326
Pulmonary hypertension	6 (3.4%)	2 (3.2%)	4 (3.5%)	*p* = 0.915	2 (4.7%)	0 (0.0%)	*p* = 0.327	0 (0.0%)	2 (8.0%)	*p* = 0.310
Esophageal varices	53 (29.8%)	19 (30.2%)	34 (29.6%)	*p* = 0.934	14 (32.6%)	5 (25.0%)	*p* = 0.543	6 (33.3%)	8 (32.0%)	*p* = 0.898

Patient and tumor characteristics are compared between bridging therapy groups in Table 2. Most patients had magnetic resonance imaging (MRI) at baseline prior to bridging therapy. Patients who underwent TACE had more and larger tumors. After bridging therapy, 88% of patients had follow-up imaging prior to transplantation with MRI or CT. Twenty (80%) patients who underwent ablation and 9 (60%) patients who underwent TACE had complete response to locoregional therapy based on mRECIST imaging criteria, which defines complete response as “Disappearance of any intratumoral arterial enhancement in all target lesions” (1).

**Table 2:** **Imaging and explant characteristics of patients with hepatocellular carcinoma**.

		All patients with HCC (*n* = 63)	TACE (*n* = 18)	Ablation	
**Imaging prior to bridging therapy**					
Imaging type	MRI	37 (86.0%)	15 (83.3%)	21 (84.0%)	*p* = 0.823
	CT	4 (9.3%)	2 (11.1%)	2 (8.0%)	
Number tumors median		1.5	1	1	*p* = 0.014
Largest tumor dimension median (cm)		2.7	2.9	2.3	*p* = 0.149
**Imaging after bridging therapy**					
Imaging type	MRI	37 (86.0%)	13 (76.5%)	23 (95.8%)	*p* = 0.134
	CT	1 (2.3%)	1 (5.9%)	0 (0.0%)	
Post-bridge imaging with HCC	Yes	9 (23.7%)	6 (40.0%)	3 (13.0%)	*p* = 0.104
	No	29 (67.4%)	9 (60.0%)	20 (87.0%)	
**Explant – patients grouped on first bridging procedure**					
Viable tumor on explant	Yes	50 (79.4%)	11 (61.1%)	21 (84.0%)	*p* = 0.069
	No	13 (20.6%)	7 (38.9%)	4 (16.0%)	
Number of tumors on explant pathology	0	3 (4.8%)	1 (5.6%)	1 (4.0%)	*p* = 0.642
	1	32 (50.8%)	6 (33.3%)	15 (60.0%)	
	2	13 (20.6%)	6 (33.3%)	5 (40.0%)	
	3	13 (20.6%)	4 (22.2%)	3 (12.0%)	
	4	2 (3.2%)	1 (5.6%)	1 (4.0%)	
Explant tumor necrosis	Yes	39 (62.9%)	12 (66.7%)	20 (80.0%)	*p* = 0.482
	No	23 (37.1%)	6 (33.3%)	5 (20.0%)	
Explant tumor necrosis, mean (SD)		34.39%	41.20%	47.00%	*p* = 0.787
Explant pathology T-stage	TO	3 (4.8%)	2 (11.1%)	1 (4.0%)	*p* = 0.198
	T1	27 (42.9%)	4 (22.2%)	13 (52.0%)	
	T2	23 (36.5%)	8 (44.4%)	6 (24.0%)	
	T3a	2 (3.2%)	0 (0.00%)	2 (8.0%)	
	None	8 (12.7%)	4 (22.2%)	3 (12.0%)	
Tumor grade	1	12 (24.5%)	2 (20.0%)	1 (4.8%)	*p* = 0.346
	2	26 (53.1%)	6 (60.0%)	13 (61.9%)	
	3	9 (18.4%)	2 (20.0%)	5 (23.8%)	
Explant largest tumor diameter in cm, mean (SD)		2.8	2.4	3.8	*p* = 0.141

The mean number of days between initial bridging therapy and transplantation was 242 (212 days for TACE patients and 249 days for ablation patients). Explant pathology characteristics are detailed in Table 2. Viable tumor was identified in explant pathology in 32 patients (74%), including 11 (61%) patients who underwent TACE and 21 (84%) patients who underwent ablation. For the 21 patients who underwent ablation and had viable tumor on explant pathology, 16 (76.2%) had viable tumor in the ablated nodule(s) and 5 (23.8%) had viable tumor in both the ablated nodule(s) and in one or more separate nodule(s).

**Figure 1:**
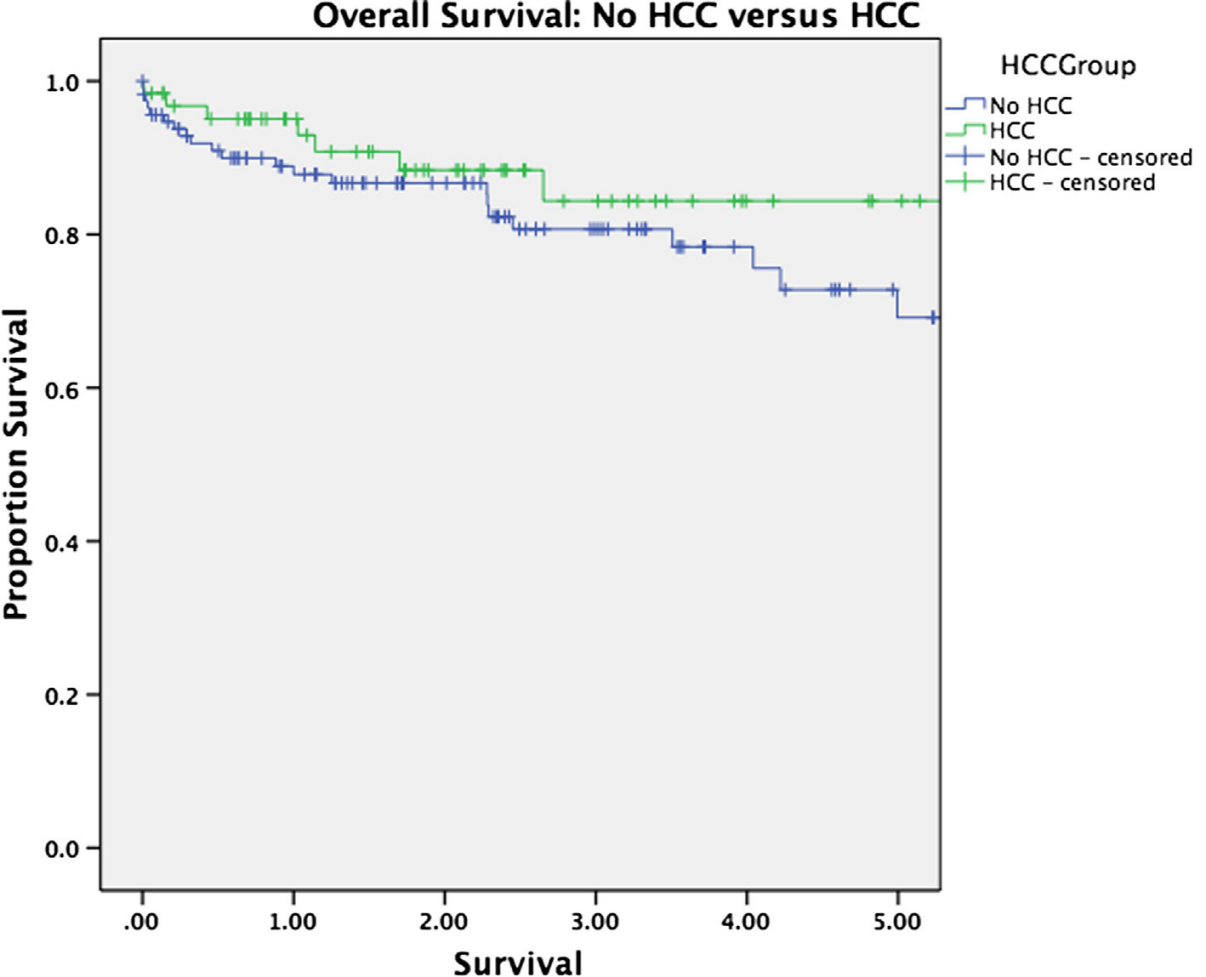
**Kaplan–Meier survival curves are shown for patients transplanted with and without HCC**.

**Figure 2:**
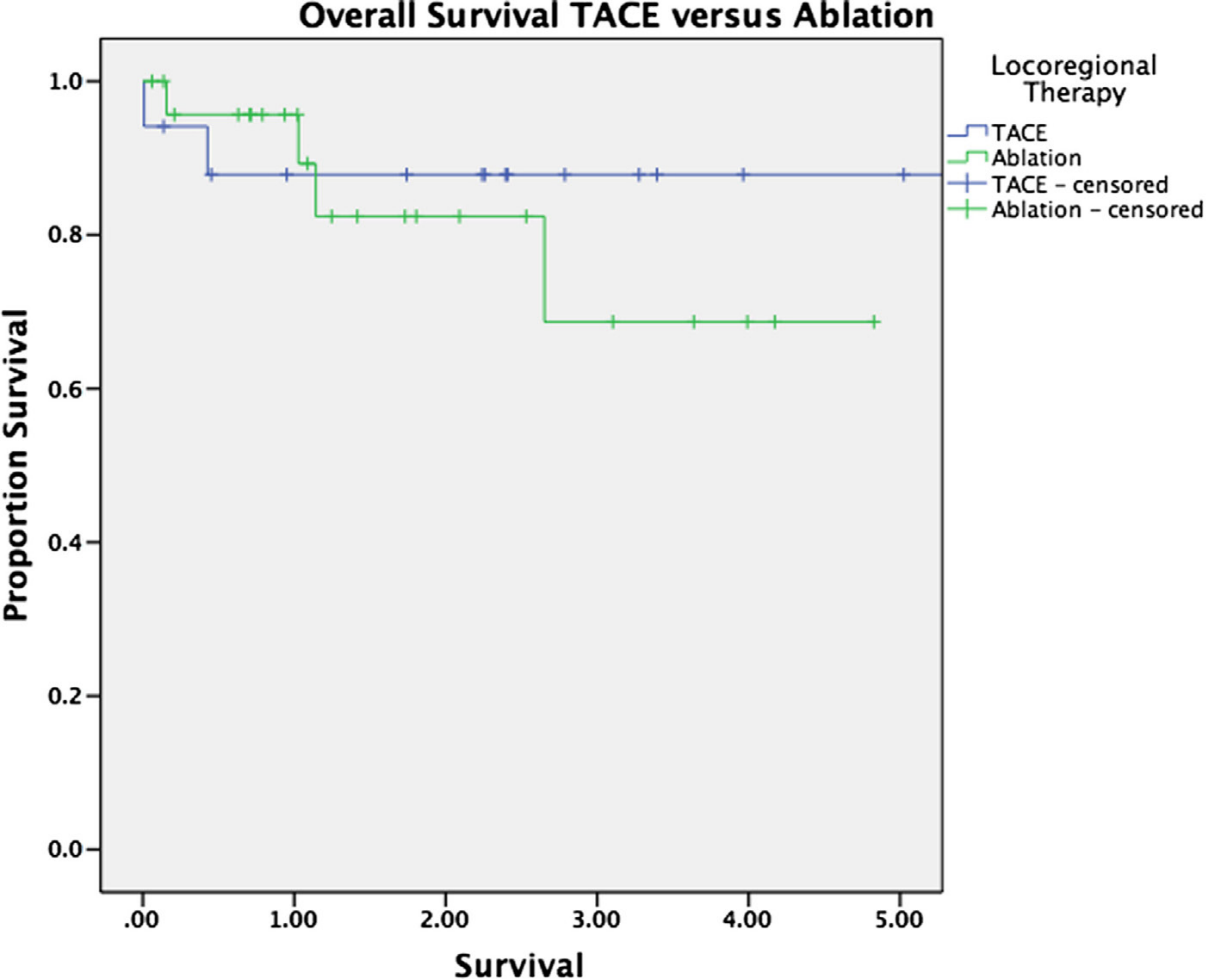
**Kaplan–Meier survival curves are shown for patients bridged with TACE and those bridged with ablation**.

**Figure 3:**
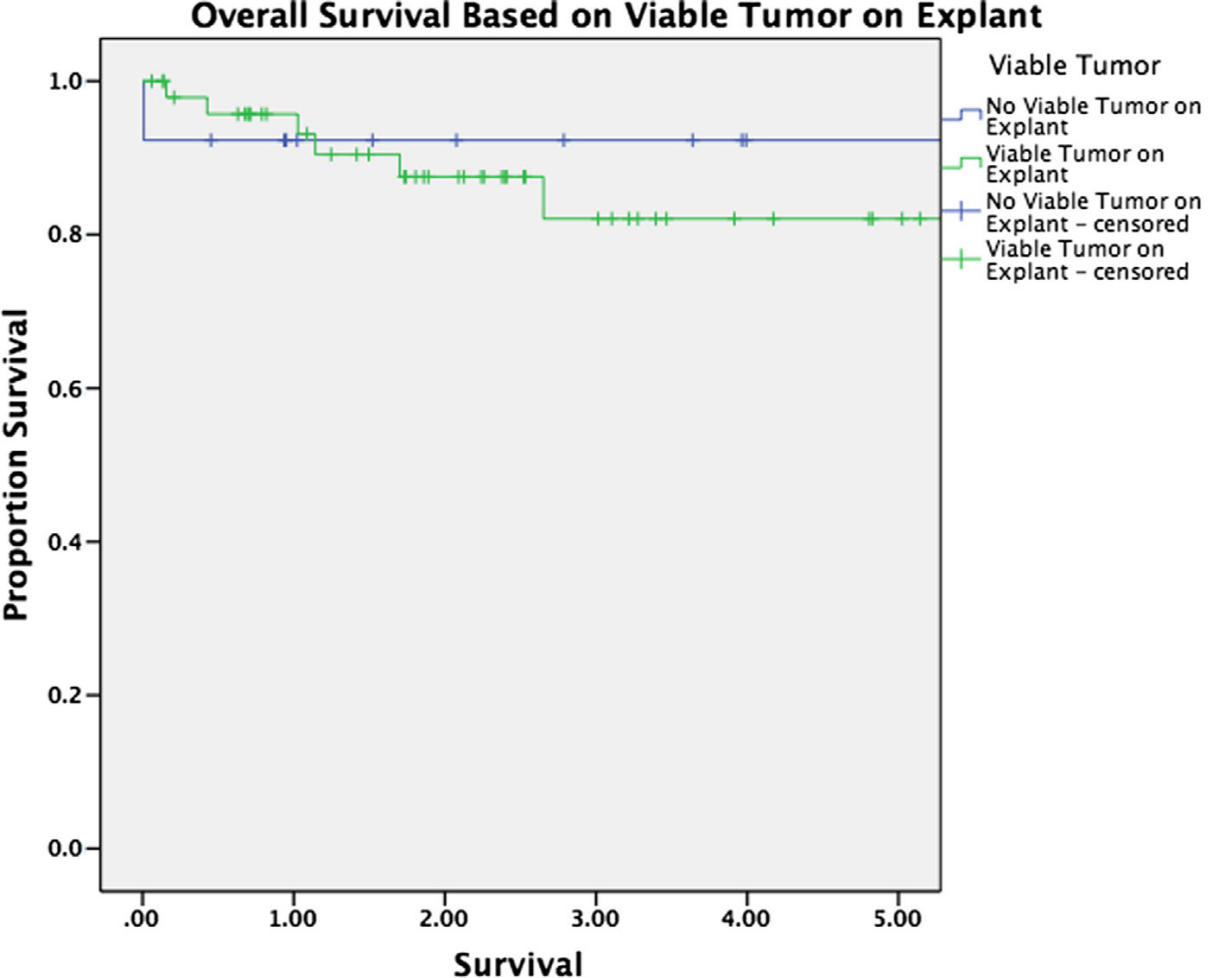
**Kaplan–Meier survival curves are shown for patients with viable tumor on explant pathology and without viable tumor on explant pathology after bridging therapy**.

Median follow-up was 30 months for the entire cohort (33 months for patients alive at last follow-up and 16 months for patients who have died). There was no significant difference in overall survival between patients with and without HCC (*p* = 0.263, Figure 1). Survival was equivalent in patients who underwent TACE versus those who underwent ablation (*p* = 0.575, Figure 2). The presence or absence of viable tumor was not associated with overall survival in the HCC cohort (Figure 3); there was one patient who experienced a recurrence during the study period. No other differences in outcomes were detected between the viable and non-viable tumor groups.

The patient who experienced recurrence was a 50-year-old male with history of abdominal gunshot wound and cirrhosis secondary to HCV and HCC. He was diagnosed with two HCC lesions in segment VI of the liver measuring 1.9 cm × 2.1 cm and 1.5 cm × 1.7 cm. He underwent CT-guided radiofrequency ablation of both lesions. Six months later, MRI demonstrated a lesion in the left hepatic lobe concerning for HCC and the patient underwent left hepatic artery TACE. He did not undergo follow-up imaging, and 1 month later, he underwent liver transplantation. He had an uneventful postoperative course. One year after transplantation, he presented with elevated liver enzymes and underwent blind follow-up liver biopsy, which demonstrated recurrent HCC. He subsequently underwent MRI, which demonstrated multifocal tumor in the liver most consistent with HCC, osseous lesions concerning for metastases, and extensive upper abdominal and retroperitoneal lymphadenopathy. The patient and his family opted for hospice care, and he passed away prior to his next follow-up appointment.

## Discussion

Rates of viable tumor in the hepatic explant were high in this series after bridging therapy with either TACE or ablation. The presence of viable tumor in the hepatic explant was not associated with worse outcome, although our numbers may be underpowered to detect a difference. Agopian et al. recently reported a large series of 501 patients who underwent locoregional therapies prior to liver transplantation at a single high-volume institution and concluded that 126 patients (25%) had complete tumor necrosis. Contrary to our results, they found that complete tumor necrosis was associated with superior outcomes. Overall survival was not reported in this study. Transplant was done for 13% of patients originally outside of Milan criteria in this series, and the overall recurrence of HCC was 15.0% in patients without complete pathologic response and 2.4% in patients with complete pathologic response (15). While the rate of recurrence in our study was lower, patient selection is the likely explanation as all patients transplanted had HCC within Milan criteria (and none were downstaged).

TACE and ablation are common bridging therapies for HCC prior to transplantation (15). Reported rates of complete tumor necrosis, or complete pathologic response, vary based on treatment modality with response rates between 0 (12) and 57% (13) reported for TACE and 8.3 (12) and 74% (14) reported for radiofrequency ablation. Generally, patients who achieve transplant after these bridging therapies have excellent outcomes with good overall and recurrence-free survival, and these therapies are well tolerated without high risk of morbidity (16, 17). Guidelines recommend that patients with HCC be considered for bridging therapy (10) if they are expected to wait more than 6 months for transplantation in order to reduce dropouts from the waitlist secondary to tumor progression (11).

This is consistent with prior studies. Marin et al. evaluated 28 patients with 38 nodules who underwent TACE, selective internal radiation therapy (SIRT), and chemical or radiofrequency ablation and noted that 42% of patients had post-bridging imaging concerning for HCC and that there was viable tumor in 35 (90%) of the 39 treated nodules on explant pathology (12). Lu et al. looked at 47 HCC nodules in 24 patients treated with single or double RFA prior to liver transplantation and concluded that 35 (74%) of the tumors were successfully treated. They determined that small lesions and non-perivascular lesions were more likely to be successfully treated with this modality and concluded that RFA is an effective treatment for small (<3 cm) HCC (14). Wong et al. evaluated patients who had undergone TACE, percutaneous ethanol injection (PEI), and radiofrequency ablation (RFA) and determined that of 44 treated nodules, 29 (66%) had a least 75% necrosis (18).

Limitations of this study include retrospective design, small sample size, and median follow-up slightly <3 years. As mentioned, this may mean that the analysis lacked power to detect an influence of tumor viability on outcome. Additionally, due to the small size of our cohort, we were not able to detect differences in other outcomes, including the quality of life. Since the number of recurrence cases of HCC is very low, we conclude that transplant after bridging therapy for patients within Milan criteria does not require serial bridging to achieve complete pathologic tumor response. The presence of complete response by imaging is adequate to allow close observation with serial imaging prior to transplant.

In conclusion, we determined that although complete pathologic response to bridging therapy is not common, this does not have an impact on transplantation outcomes. Alternate methods may be required to determine if tumor cells that appear viable on pathologic analysis are actually alive. Further studies in this area could include assessing whether bridged cells are less likely to grow in culture or in xenografts in comparison to non-bridged (incidental) tumors.

## Author Contributions

EB, CS, and MD were responsible for initial study design. EB and KD collected data and performed the data analysis. EB, KD, AH, AM, LC, GD, SB, PB, MD, and CS performed interpretation of the data, composed the manuscript, and provided critical review and revision.

## Conflict of Interest Statement

The authors declare that the research was conducted in the absence of any commercial or financial relationships that could be construed as a potential conflict of interest.
